# Metabolomics profiling reveals distinct, sex‐specific signatures in serum and brain metabolomes in mouse models of Alzheimer's disease

**DOI:** 10.1002/alz.13851

**Published:** 2024-04-27

**Authors:** Ravi S. Pandey, Mattias Arnold, Richa Batra, Jan Krumsiek, Kevin P. Kotredes, Dylan Garceau, Harriet Williams, Michael Sasner, Gareth R. Howell, Rima Kaddurah‐Daouk, Gregory W. Carter

**Affiliations:** ^1^ The Jackson Laboratory for Genomic Medicine Farmington Connecticut USA; ^2^ Department of Psychiatry and Behavioral Sciences Duke University Durham North Carolina USA; ^3^ Institute of Computational Biology Helmholtz Zentrum München German Research Center for Environmental Health, Ingolstädter Landstraße 1 Oberschleißheim Germany; ^4^ Department of Physiology and Biophysics Institute for Computational Biomedicine Englander Institute for Precision Medicine Weill Cornell Medicine New York New York USA; ^5^ The Jackson Laboratory Bar Harbor Maine USA; ^6^ Duke Institute of Brain Sciences Duke University Durham North Carolina USA; ^7^ Department of Medicine Duke University Durham North Carolina USA

**Keywords:** 5XFAD, Alzheimer's disease, animal models, apolipoprotein E, metabolomics

## Abstract

**INTRODUCTION:**

Increasing evidence suggests that metabolic impairments contribute to early Alzheimer's disease (AD) mechanisms and subsequent dementia. Signals in metabolic pathways conserved across species can facilitate translation.

**METHODS:**

We investigated differences in serum and brain metabolites between the early‐onset 5XFAD and late‐onset LOAD1 (APOE4.Trem2*R47H) mouse models of AD to C57BL/6J controls at 6 months of age.

**RESULTS:**

We identified sex differences for several classes of metabolites, such as glycerophospholipids, sphingolipids, and amino acids. Metabolic signatures were notably different between brain and serum in both mouse models. The 5XFAD mice exhibited stronger differences in brain metabolites, whereas LOAD1 mice showed more pronounced differences in serum.

**DISCUSSION:**

Several of our findings were consistent with results in humans, showing glycerophospholipids reduction in serum of apolipoprotein E (apoE) ε4 carriers and replicating the serum metabolic imprint of the *APOE* ε4 genotype. Our work thus represents a significant step toward translating metabolic dysregulation from model organisms to human AD.

**Highlights:**

- This was a metabolomic assessment of two mouse models relevant to Alzheimer's disease.
- Mouse models exhibit broad sex‐specific metabolic differences, similar to human study cohorts.
- The early‐onset 5XFAD mouse model primarily alters brain metabolites while the late‐onset LOAD1 model primarily changes serum metabolites.
- Apolipoprotein E (apoE) ε4 mice recapitulate glycerophospolipid signatures of human *APOE* ε4 carriers in both brain and serum.

## 1 BACKGROUND

Alzheimer's disease (AD) is the leading cause of dementia, characterized by the accumulation of amyloid plaques and tau fibrillary tangles in the brain.^1^, ^2^, ^3^ Early‐onset Alzheimer's disease (EOAD, familial AD or FAD) is often caused by mutations in genes coding for amyloid precursor protein (*APP*), presenilin 1 (*PSEN1*), and presenilin 2 (*PSEN2*).^4^, ^5^ Sporadic or late‐onset AD (LOAD) is more common (> 95% prevalence), with symptoms arising after age 65.^5^, ^6^ Its risk factors include age, the apolipoprotein E (*APOE*) ε4 allele, and point mutations in triggering receptor expressed on myeloid cells 2 (*TREM2*).^3^, ^7^, ^8^, ^9^, ^10^ Females are at higher risk^11^, ^12^ and comprise the majority of cases,^13^ and female *APOE* ε4 carriers are at greater risk of developing AD compared to male carriers.^14^, ^15^, ^16^

Pathophysiological changes associated with AD begin decades before clinical symptoms.^11^ Metabolic decline is among the earliest symptoms, with reduced glucose uptake in patients with mild cognitive impairment (MCI).^17^ Furthermore, disruption in glucose metabolism is associated with early mitochondrial dysfunction in animal models and AD‐affected individuals.^18^ Perturbations in multiple metabolic networks such as lysine metabolism, tricarboxylic acid cycle, and lipid metabolism were reported in MCI individuals compared to healthy individuals.^19^ This suggests that metabolic dysfunction could play an important role in early disease stages.

Metabolic dysregulation is one of the hallmarks of AD, and is known to result from three major AD risk factors: age, *APOE* genotype in European cohorts, and sex.^20^, ^21^, ^22^, ^23^ A recent study in the Alzheimer's Disease Neuroimaging Initiative (ADNI) identified the effects of sex and *APOE* ε4 on metabolic alterations and AD biomarkers.^12^ Even in mouse models of AD, changes in metabolic pathways related to energetic stress were more pronounced in female mice compared to males.^19^, ^24^ However, the molecular mechanisms underlying these sex‐linked differences remain undetermined.

Heterogeneity in humans complicates the molecular study of disease mechanisms.^25^ For human studies, experimental design is often limited by sample availability, reducing confidence in inferences.^11^ Animal models have been critical for understanding the development and progression of AD and enable the study of disease‐related risk factors in a controlled environment.^3^, ^25^ Inbred mouse models of AD facilitate the collection of cross‐sectional sampling at multiple ages and analysis of metabolic changes during various life stages. Signals in conserved metabolic pathways could thus provide a method to translate experimental findings in preclinical mouse models to humans.^11^ Transgenic mouse models with gene mutations for *APP* and *PSEN1* were widely used to investigate the biofluids and brain metabolome and observed significant overlap in the affected metabolic pathways identified in AD patients.^26^, ^27^, ^28^ However, these mouse models were limited to fAD transgenic models that represent a small number of AD cases, and previous studies did not interrogate the influence of sex‐specific differences in metabolic changes.

To fill this gap, we comprehensively profiled the serum and brain metabolomes of APOE4.Trem2*R47H mice (a genetic model for LOAD), the 5XFAD mice (a transgenic amyloid model), and C57BL/6J (control) mice at 6 months of age in both sexes. A total of 142 metabolites were measured in both brain and serum, including glycerophospholipids, sphingolipids, amino acids, biogenic amines, and acylcarnitines. The LOAD1 strain carries two primary risk alleles, humanized *APOE* ε4/ε4 and the homozygous Trem2*R47H variant on the C57BL/6J (B6) background.^29^ The 5XFAD transgenic mice overexpress human *APP* with three FAD mutations and human *PSEN1* with two FAD mutations on the B6 background.^30^ We investigated the sex differences in metabolic effects for the 5XFAD and the *APOE* ε4/ε4 genotype in both blood and serum metabolomes as well as the correspondence between brain and serum metabolite levels in mouse models. We compared our findings to recent results from ADNI^12^ and Rush Religious Order Study and Memory and Aging Project (ROS/MAP) cohorts.^31^ All datasets described in this study are available through the AD Knowledge Portal (https://adknowledgeportal.synapse.org/).

## 2 METHODS AND MATERIALS

### 2.1 Animal models

All animal models were obtained from The Jackson Laboratory. All experiments were approved by the Animal Care and Use Committee at The Jackson Laboratory. LOAD1 mice (JAX #28709) carry a humanized version of the prominent *APOE* ε4/ε4 genetic risk factor for LOAD, and a relatively rare deleterious variant R47H allele of the Trem2 gene,^29^ while the 5XFAD transgenic mice (JAX #8730) overexpress five FAD mutations: the APP(695) transgene contains the Swedish (K670N, M671L), Florida (I716V), and London (V7171) mutations and the PSEN1 transgene contains the M146L and L286V FAD mutations.^30^, ^32^ Cohort of male and female LOAD1, 5XFAD, and C57BL/6J (B6) control mice were assayed for serum and brain metabolomics at 6 months of age (Table 1).

**TABLE 1 alz13851-tbl-0001:** Number of biological replicates for mouse serum and brain tissue metabolomics.

	Female	Male
5XFAD	12	13
LOAD1	13	10
C57BL/6J	14	12

*Note*: Serum and brain samples were obtained from the same mice.

### 2.2 Metabolomics data acquisition and processing

A uniform technical approach was used for both mouse and human metabolomics processing. Full sample preparation and analysis protocols are described on the AD Knowledge Portal (https://adknowledgeportal.synapse.org/Explore/Studies/DetailsPage?Study=syn22313528). In brief, metabolites were measured with the targeted AbsoluteIDQ‐p180 metabolomics kit (Biocrates Life Sciences AG), with an ultra‐performance liquid chromatography‐tandem mass spectrometry (MS/MS) system (Acquity UPLC [Waters], TQ‐S triple quadrupole MS/MS [Waters]), which provides measurements of up to 186 endogenous metabolites. Sample extraction, metabolite measurement, identification, quantification, and primary quality control (QC) followed standard procedures.^33^ In the serum metabolome, 27 metabolites were removed due to missing values in > 20% of samples and four metabolites were excluded due to > 20% coefficient of variation. In the brain metabolome, 20 metabolites were removed due to missing values and one metabolite was excluded due to higher coefficient of variation. After median‐based batch correction, metabolite concentrations were log2‐transformed and missing values for 79 serum metabolites and seven brain metabolites were imputed using kNN (k‐nearest‐neighbor, k = 10) method.^34^

### 2.3 ADNI and ROS/MAP metabolomics data

Processing of ADNI[Table alz13851-tbl-0001] P180 measurements has been described in detail.^12^ In brief, metabolites with > 20% missing values were excluded and batch correction was performed using a cross‐plate mean normalization procedure using National Institute of Standards and Technology standard plasma metabolite concentrations. Metabolites having a coefficient of variation > 20% or an intraclass correlation < 65% in replicate samples were removed. Missing values were imputed using minimum imputation (set to half of the plate‐specific lower limit of detection). Metabolite levels were log2‐transformed and multivariate sample outliers were excluded. Finally, metabolites were adjusted for significant medication effects using stepwise backward selection.

In ROS/MAP, brain and serum metabolites and samples with > 25% missing values were filtered out. Quotient normalization was used to correct for sample‐wise variation across the metabolites.^35^ Metabolite values were subsequently log2‐transformed, and the remaining missing values were imputed using kNN as for the mouse data. Data processing was performed using standardized pipelines in the toolbox maplet.^36^

Mouse experiments were carried out following the human assays in both ADNI and ROS/MAP, generated using the Biocrates AbsoluteIDQ‐p180 metabolomics kit. This ensured an identical targeted quantification across species.

**RESEARCH IN CONTEXT**

1. **Systematic review**: The authors reviewed the literature using traditional (e.g., PubMed) sources and meeting abstracts and presentations. Several recent publications have suggested metabolic dysregulation as a major hallmark in early stages of Alzheimer's disease (AD). The relevant studies are appropriately cited.
2. **Interpretation**: Our findings indicate that metabolomic signatures were notably different between brain and serum in the mouse models of AD. We were able to identify patterns of metabolic changes related to human AD risk in our mouse models, which strengthen the translational utility of mouse models for studying metabolic changes that appear in early‐stage AD.
3. **Future directions**: The article proposes to investigate metabolomics profiles of the late‐onset mouse models over different extended ages to study progressive metabolic changes associated with AD pathology. Our findings suggest such aging studies can model the different phases of metabolic disturbances that may occur in human AD.

### 2.4 Principal component analysis

We analyzed a total of 142 metabolites present in both serum and brain metabolome from 74 samples originating from different mouse models. We extracted the principal components using singular value decomposition for both metabolomes separately and plotted results using the ggplot2 visualization package in R.

### 2.5 Association analyses for the mouse metabolomic data

Association analyses of AD risk factors with metabolite levels were conducted using standard linear regression. The stratification variable sex was excluded as a covariate in the sex‐stratified analyses. We transformed covariate‐adjusted effect sizes to sample size‐weighted standardized effects (Cohen *d*). For identifying metabolic sex differences, we used linear regression with metabolite levels as the dependent variable and sex as explanatory variable. To adjust for multiple testing, we used the threshold of Bonferroni significance of 9.09 × 10^−4^ as determined in a recent study.^12^ We performed two‐sample *t* tests to identify significant sexual dimorphisms for metabolites with the standardized effect sizes observed in the pooled mouse samples at Bonferroni significance in B6, LOAD1, and 5XFAD mouse models. To assess the significance of heterogeneity between strata, we used methodology similar to the determination of study heterogeneity in an inverse‐weighted meta‐analysis.^37^ We further provide a scaled (0%–100%) index of percent heterogeneity, similar to the *I* ^2^ statistic.

### 2.6 Association analysis for the ADNI cohort

Association analysis of the cerebrospinal fluid (CSF) amyloid beta (Aβ)_1‐42_ pathology with blood metabolite levels from the ADNI cohort followed published protocols in Arnold et al.^12^ with a few adjustments. We performed the multivariable linear regression analysis using metabolite concentrations as the dependent variable to test associations of the CSF Aβ_1‐42_ pathology with concentrations of 139 blood metabolites in place of the logistic regression approach used by Arnold et al.,^12^ ensuring consistency with the approach used for mouse metabolomics data.

### 2.7 Correlation analysis

First, we performed the multivariable linear regression analysis using metabolite concentrations of 70 glycerophospholipids (PC species) as dependent variables to determine the effect of *APOE* ε4 in serum metabolomics data from mouse, ADNI, and ROS/MAP carriers. We also fit a multivariable linear regression model for brain metabolomics data from mouse models and ROS/MAP individuals to measure the effect of *APOE* ε4 on glycerophospholipids (PCs) levels. For serum and brain metabolomics data from mouse models, we used the 5XFAD genotype and male sex as a covariate. For serum metabolomics data from the ADNI cohort, we used pathological CSF Aβ_1‐42_, age, male sex, cohort, and body mass index (BMI) as covariates. For serum metabolomics data from the ROSMAP cohort, we used pathological amyloid levels, fasting status, age at visit, sex, education, and BMI as covariates. For brain metabolomics data from the ROS/MAP cohort, we used pathological amyloid levels, age, sex, education, and BMI as covariates. Finally, we measured the Pearson correlation between effects of *APOE* ε4 on glycerophospholipids (PCs) metabolite levels in human and mouse models. We also measured Pearson correlations between the effects of *APOE* ε4 presence on glycerophospholipids (PCs) metabolite levels in mouse serum and brain metabolomes.

## 3 RESULTS

### 3.1 Sex is a separator in both serum and brain metabolome

**FIGURE 1:**
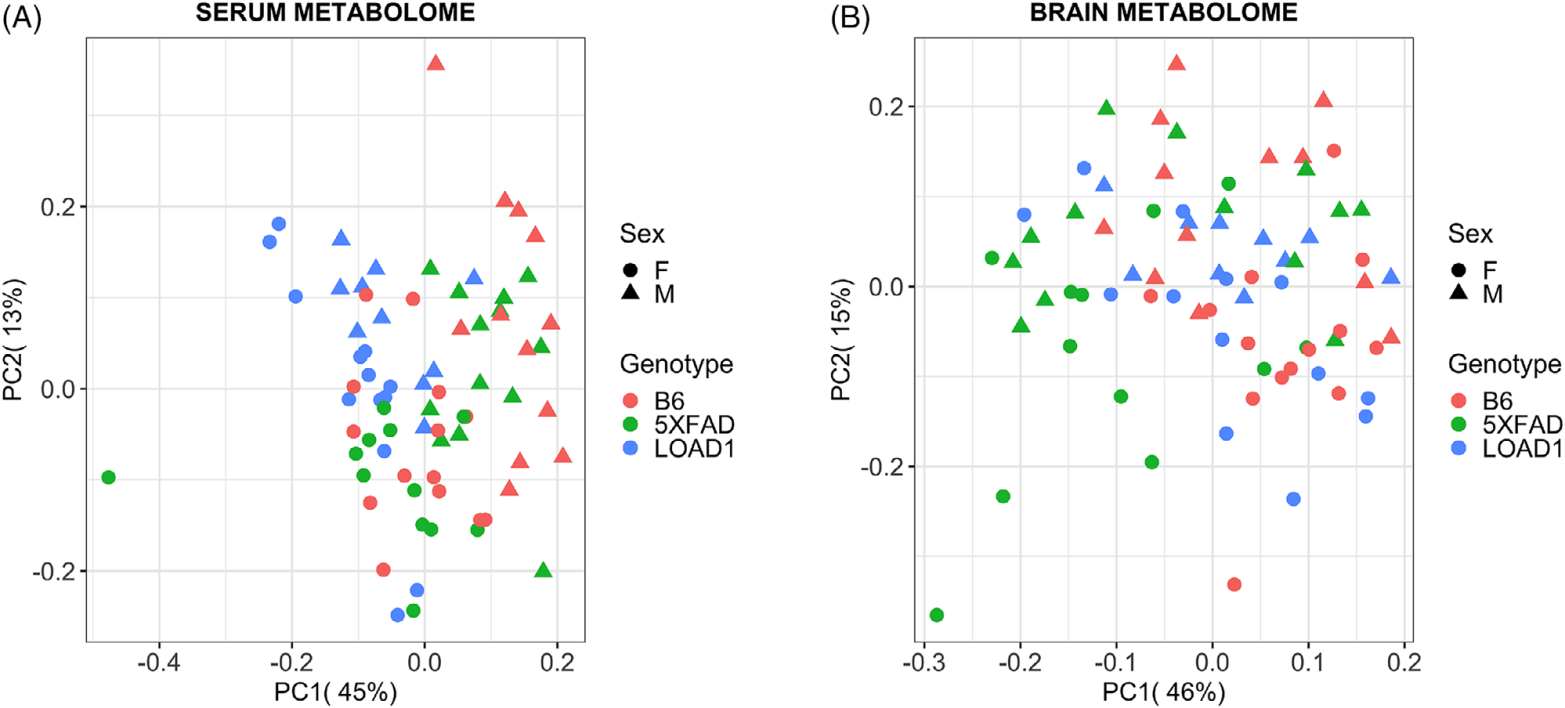
Principal components analysis of serum and brain metabolomes from mouse models of Alzheimer's disease. A, In the serum metabolome, we observed a slight gradient of discrimination of the LOAD1 samples from B6 and 5XFAD samples along the second principal component. B, In the brain metabolome, we found a gradient of discrimination of the 5XFAD samples from B6 and LOAD1 samples along the first principal component.

Principal component analysis indicated serum metabolites separated samples by sex of the mice along the first principal component (45% of total variance), whereas we observed a slight gradient of discrimination by genotype along the second principal component (13% of total variance; Figure 1A). Specifically, LOAD1 mice were segregated from B6 and 5XFAD mice. In brain metabolomes, principal component analysis did not reveal any clear separation between groups (sex or genotype). However, a gradient of discrimination separated 5XFAD samples from B6 and LOAD1 samples along the first principal component (46% of total variance), whereas most of the male and female samples were separated along the second principal component (Figure 1B). Overall, this suggested global sex‐specific differences in both serum and brain metabolomes.

### 3.2 Sex‐associated differences significantly differ in AD mouse models

Next, we investigated sex‐specific associations of metabolites in all mice together, as well as in each mouse model (B6, LOAD1, and 5XFAD) separately. We then tested whether these sex‐specific differences were altered in LOAD1 and 5XFAD mice compared to B6 mice to determine strain‐specific sex differences.

Throughout this article, metabolites belonging to glycerophospholipid (phosphatidylcholines and lyso‐phosphatidylcholines) class will be referred to as PC. In addition, ethers containing glycerophospholipids (PCs) will be denoted by PC ae Cx:z, diacyl‐PCs will be denoted by PC aa Cx:z, and acyl containing lyso‐phosphatidylcholines will be denoted as lysoPC a Cxy:z. Sphingomyelins will be denoted as SM Cxy:z and hydroxy sphingomyelins will be denoted as SM (OH) Cxy:z. Metabolites belonging to acylcarnitines class will be denoted as Cx or Cxy:z.

#### 3.2.1 Mouse serum metabolome

**FIGURE 2:**
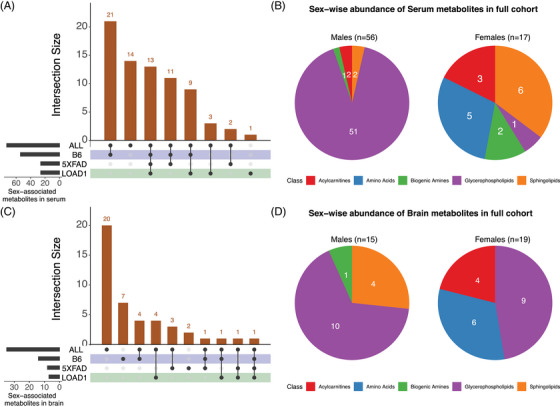
Sex‐metabolite associations in mouse models. We identified metabolites significantly associated with sex in the full mouse cohort after multiple testing corrections (*P* < 9.09 × 10^−4^) while adjusting for genotypes and each of the three strains (B6, LOAD1, and 5XFAD). A, An UpSet plot showing intersection between sex‐associated serum metabolites across full cohort (ALL) and each of the three strains. Horizontal bars on the left represent number of metabolites significantly associated with sex and vertical bars represent number of sex‐associated metabolites shared across full cohort and each of the three genotypes. Each black dot represents the group and connecting lines represent the group being compared. In serum metabolome, we found 73 out of 142 metabolites significantly associated with sex in the full cohort. Every significant sex difference found in each genotype alone was also significant in full cohort except one that showed significant sex difference in only LOAD1 genotype. B, Pie charts representing classes of altered metabolites in each sex in the full cohort for serum metabolome. Colors in each chart represents distinct classes of metabolites (red: acylcarnitines, blue: amino acids, green: biogenic amines, purple: glycerophospholipids, and orange: sphingolipids). The majority of glycerophospholipids were more abundant in male mice, while amino acids and sphingolipids were more abundant in female mice. C, An UpSet plot showing intersection between sex‐associated brain metabolites across full cohort and each of the three strains. In the brain metabolome, we found 34 out of 142 metabolites to be significantly associated with sex in the full cohort. D, Pie charts representing classes of abundant metabolites in each sex in the full cohort. The sphingolipids were more abundant in male mice, while amino acids and acylcarnitines were more abundant in female mice.

In the complete cohort (*N* = 74), 73 out of 142 metabolites were significantly associated with sex after multiple testing corrections (*P* < 9.09 × 10^−4^) while adjusting for genotypes (Figure 2A, Table S1 in supporting information). Fifty‐six of these metabolites had higher levels in males, and 17 metabolites had higher levels in females (Figure 2B). The majority of glycerophospholipids (PCs) were more abundant in male mice, while levels of a few amino acids (alanine, isoleucine, serine, and threonine) and the majority of sphingolipids (SMs) were more abundant in female mice (Figure 2B, Table S1). Notably, 54 of these sex‐specific associations were also observed in a recent study from the ADNI cohort.^12^ Further, stratification by genotypes revealed that 13 of the 73 metabolites showing significant sex differences in the complete cohort were also significant in each of the three genotypes (B6, LOAD1, and 5XFAD) separately, whereas 14 metabolites showed no significant difference in any genotype (Table S1, Figure 2A). Significant sex differences limited to one genotype were found for three metabolites (lyso‐phosphatidylcholine acyl C20:4 [lysoPC a C20:4], SM [OH] C24:1, asymmetric dimethylarginine [ADMA]) in LOAD1 mice, for two metabolites (C3‐DC [C4‐OH], PC ae C30:1) in the 5XFAD mice, and for 21 metabolites in B6 mice. Every significant sex difference found in each genotype alone was also significant in the full cohort except one: PC aa C38:4 showed a significant sex difference only in the LOAD1 genotype, with higher levels in females (Table S1).

Further, comparisons of beta estimates for sex between 5XFAD and B6 groups showed significant effect heterogeneity (*P*_HET_ < 0.05) for five metabolites (C18:1, C18:2, PC aa C34:2, PC aa C36:2, asymmetric dimethylarginine [SDMA]). All five metabolites reversed their direction of abundance change between sexes in the 5XFAD mice compared to B6 mice. Notably, none of these metabolites showed significant sex‐specific differences either in combined mouse samples or separated by genotypes. Similarly, comparisons of beta estimates for sex between LOAD1 and B6 groups showed significant effect heterogeneity (*P*_HET_ < 0.05) for 31 metabolites, out of which 23 were glycerophospholipids and three were amino acids (valine, arginine, and phenylalanine; Table S2 in supporting information). Most of these metabolites had opposite changes in direction between sexes in LOAD1 compared to B6 controls. These observations indicated that sex differences in serum metabolite levels were significantly affected in the late‐onset AD model compared to control mice.

#### 3.2.2 Mouse brain metabolome

Next, we investigated sex‐specific associations of metabolites and sex‐associated differences in brain metabolomes of mouse models. In the complete cohort (*N* = 74), 34 out of 142 metabolites were significantly associated with sex after multiple testing correction (P < 9.09 × 10^−4^) when adjusting for genotypes (Figure 2C, Table S1). A total of 15 metabolites had higher levels in males including 10 glycerophospholipids, 4 sphingolipids (SMs), and 1 biogenic amine, while 19 metabolites had higher levels in females including 9 glycerophospholipids, 4 amino acids, and 6 acylcarnitines (Figure 2D, Table S1). Stratification by genotypes revealed 14 of the 34 metabolites with significant sex differences were significant in at least one of the genotypes (B6, LOAD1, and 5XFAD) when analyzed separately, whereas 20 showed no significant sex difference in any genotype. Significant sex differences limited to one genotype were found for four metabolites (C18:1, PC aa C32:3, PC ae C36:4, t4‐OH‐Pro) in LOAD1 mice, for three metabolites (C18, PC aa C40:4, PC ae C36:5) in the 5XFAD mice, and for four metabolites (PC ae C42:2, SM C24:0, SM [OH] C22:1, SM [OH] C24:1) in the B6 mice. Significant sex differences for two metabolites (spermidine and spermine) in 5XFAD and seven metabolites (PC ae C30:0, PC ae C30:1, PC ae C30:2, PC ae C34:0, PC ae C36:0, SM C16:0, and SM C18:0) in B6 were not significant in the full cohort (Table S1, Figure 2C).

Comparisons of beta estimates for sex between 5XFAD and B6 groups showed significant effect heterogeneity (*P*_HET_ < 0.05) for 29 metabolites, which include 21 PCs (most of which were ester‐containing PCs), 5 SMs, and 3 biogenic amines (Table S3 in supporting information). All but two metabolites showed a change in direction of abundance between sex (higher levels in females compared to males) in the 5XFAD mice compared to B6 mice. Similarly, comparisons of beta estimates for sex between LOAD1 and B6 groups showed significant effect heterogeneity (*P*_HET_ < 0.05) for 13 metabolites, which included 9 glycerophospholipids (the majority of which were ether‐containing PCs), 2 long‐chain acylcartines (C16:1, C18:2), 1 biogenic amine (SDMA), and 1 hydroxy‐SM (SM [OH] C22:1; Table S3). All but three metabolites exhibited a change in direction of abundance between sexes (higher levels in females compared to males) in LOAD1 mice compared to B6 mice. Moreover, three metabolites (PC aa C38:6, PC ae C32:1, PC ae C44:6) showed significant heterogeneity in both 5XFAD and LOAD1 mouse models compared to controls. In summary, we found that sex differences in brain metabolite levels varied by genotype in mouse models, with more pronounced effects in 5XFAD.

Moreover, we identified 19 metabolites that showed sex‐specific differences in both serum and brain metabolomes in the combined analysis of all mice. Notably, none of the metabolites showed sex differences in both serum and brain metabolomes of the LOAD1 mice, while only one metabolite, PC aa C40:4, showed sex differences in both serum and brain metabolomes of the 5XFAD mice. Levels of PC aa C40:4 were higher in males and females in serum and brain metabolomes, respectively.

### 3.3 Sex‐stratified associations of metabolites with AD risk factors

Next, we investigated the association of metabolites with AD risk factors and whether sex modifies the associations between AD risk factors and metabolite concentrations. We tested for associations of 5XFAD and LOAD1 genotypes with concentrations of each of the 142 metabolites in both serum and brain metabolomes from the same 74 individual animals. We did this in the full cohort and separately in each sex using multivariable linear regression, followed by analysis of heterogeneity of effects between sexes. All metabolite–genotype associations were deemed significant, which fulfilled at least one of the three criteria as described in Arnold et al.^12^: (1) associations Bonferroni significant (at a threshold of *P* < 9.09 × 10^−4^) in the full cohort, (2) associations Bonferroni significant in one sex, (3) associations that showed suggestive significance (*P* < 0.05) in one sex coupled with significance for effect heterogeneity between female and male effect estimates. These significant associations were further classified into homogeneous (metabolites with similar effects in their association to the risk factors for both sexes), heterogeneous (metabolites with different effects in both sexes leading to significant heterogeneity), and sex‐specific effects (effects that were Bonferroni significant in only one sex with significant effect heterogeneity between males and females). We compared our findings in mouse models to recent results from the ADNI cohort,^12^ and therefore examined the association of metabolites with each genotype for our mice cohort using similar methods.

#### 3.3.1 Mouse serum metabolome

**FIGURE 3:**
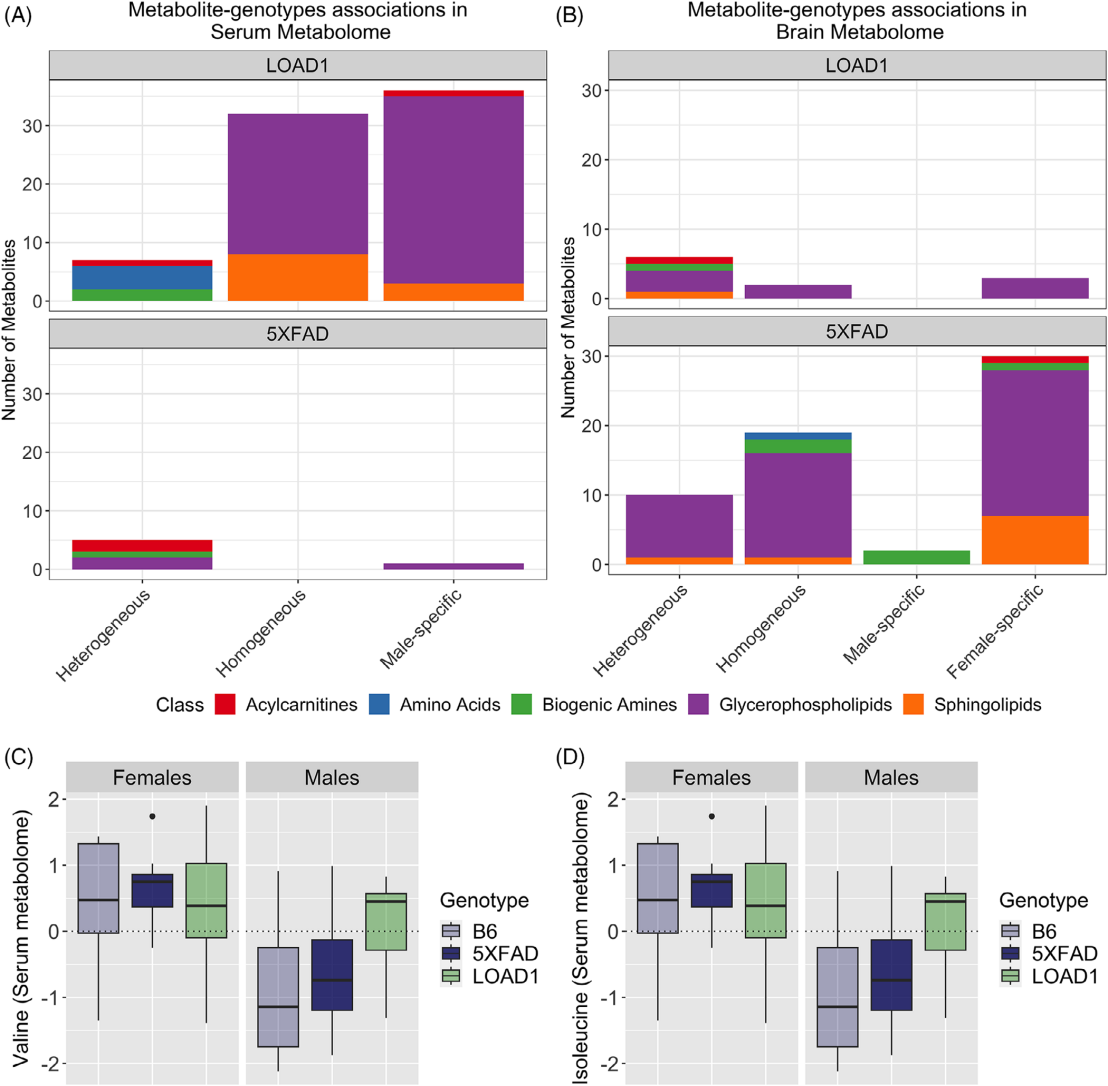
Metabolite associations with LOAD1 and 5XFAD genotype stratified by sex in mouse models. Bar charts (A‐B) showing classification of significant metabolite–genotype associations into three types of effects: homogeneous (metabolites with similar effects in their association to the risk factors for both sexes), heterogeneous (metabolites with different effects in both sexes leading to significant heterogeneity), and sex‐specific effects (effects that were Bonferroni significant [*P* < 9.09 × 10^−4^] in only one sex with significant effect heterogeneity between males and females). Different colors in the bar chart represent distinct classes of metabolites (acylcarnitines: red, amino acids: blue, biogenic amines: green, glycerophospholipids: purple, and sphingolipids: orange). A, Distribution of serum metabolites significantly associated with LOAD1 (top panel) and 5XFAD (bottom panel) strains. In serum, we identified more metabolites significantly associated with LOAD1 than 5XFAD. B, Distribution of brain metabolites significantly associated with LOAD1 and 5XFAD genotypes. In the brain metabolome, we identified more metabolites significantly associated with 5XFAD genotype then LOAD1 genotype. C‐D, Boxplots showing levels of valine and isoleucine levels for stratification by sex across each of the three strains (B6, LOAD1, and 5XFAD). Male and female groups are plotted in separate panels, with strains distinguished by color (light blue: B6, dark blue: 5XFAD, green: LOAD1). Levels of these amino acids in serum metabolome were significantly higher (*P* < 0.05) in male LOAD1 compared to male B6 controls.

In the mouse serum metabolome, we identified 83 metabolites significantly associated with the LOAD1 genotype, while only 7 metabolites were significantly associated with the 5XFAD genotype (Table S4 in supporting information, Figure 3A). Among the LOAD1 genotype‐associated metabolites, we found 32 metabolites (24 PCs and 8 SMs) with homogeneous associations (Figure 3A, top panel). Next, we identified seven associations with heterogeneous effects: one acylcarnitine (C16‐OH), one biogenic amine (alpha.AAA), and four amino acids (isoleucine, phenylalanin, tyrosine, and valine) have larger effect size in males, whereas ADMA showed stronger associations with females (all *I* ^2^ > 50%). Further, male‐specific effects were seen for 36 metabolites associated with LOAD1, which included 32 PCs, 3 SMs, and 1 acylcarnitine (C14:1‐OH) (Figure 3A, top panel). Overall, serum levels of these significant metabolites were reduced in LOAD1 mice compared to B6 controls, except for the four amino acids (isoleucine, phenylalanin, tyrosine, and valine) and one biogenic amine (alpha.AAA).

For 5XFAD, we found five metabolites with heterogenous effects (Figure 3A, bottom panel): acylcarnitines (C18:1, and C18:2) showed stronger associations with females, whereas PC aa C34:2, PC aa C36:2, and one biogenic amine (SDMA) yielded stronger associations with males (all *I* ^2^ > 50%). One male‐specific effect was seen for PC ae C38:1 with significant negative association with 5XFAD.

Another metabolite (PC aa C32:1) met the Bonferroni threshold for males but failed to meet the threshold for sex effect heterogeneity. Serum levels of these 5XFAD‐associated metabolites were also reduced in 5XFAD mice compared to B6 controls, except for one biogenic amine (SDMA). Further, we did not observe female‐specific effects for any metabolite associated with either genotype.

#### 3.3.2 Mouse brain metabolome

In the mouse brain metabolome, we identified 12 metabolites significantly associated with LOAD1, while 65 metabolites were significantly associated with the 5XFAD genotype (Table S4, Figure 3B). Out of 12 metabolites associated with LOAD1 (Figure 3B, top panel), two had homogenous effects (PC aa C42:4, PC ae C34:3) with strong positive associations. Next, we identified six associations with heterogeneous effects: acylcarnitine (C18:2) and biogenic amine (SDMA), with larger effect size in males, whereas PCs (PC aa C28:1, PC ae C40:1, PC ae C44:6) and one hydroxy sphingolipid (SM [OH] C22:1) showed stronger positive association with females (all *I* ^2^ > 50%). Further, female‐specific effects were seen for three ether‐containing PCs (PC ae C32:1, PC ae C34:0, PC ae C36:0), with significant positive correlations with the LOAD1 genotype. Levels of these significant metabolites with homogenous and female‐specific effects were increased in LOAD1 mice compared to B6 controls, while levels of metabolites with heterogenous effects were reduced in males and increased in females. Overall, we observed increased levels of these metabolites in female LOAD1 mice compared to female B6.

Out of 65 metabolites associated with the 5XFAD genotype (Figure 3B, bottom panel), we found 19 metabolites with homogenous effects, which includes 15 PCs, 1 SM (SM C16:1), 1 amino acid (lysine), and 2 biogenic amines (creatinine and t4‐OH‐Pro). Six PCs and one hydroxy sphingolipid (SM [OH] C22:2) showed heterogenous effects with stronger positive associations with females (*I* ^2^ > 50%). We also identified 30 metabolites with female‐specific effects: 21 PCs, 7 SMs (including four hydroxy SMs), 1 biogenic amine (putrescine), and 1 acylcarnitine (C18; Figure 3B, bottom panel). Levels of these 5XFAD‐associated metabolites were higher in 5XFAD mice compared to B6 controls. Male‐specific effects were also seen for two biogenic amines (spermidine, spermine), with strong negative associations with 5XFAD.

**FIGURE 4:**
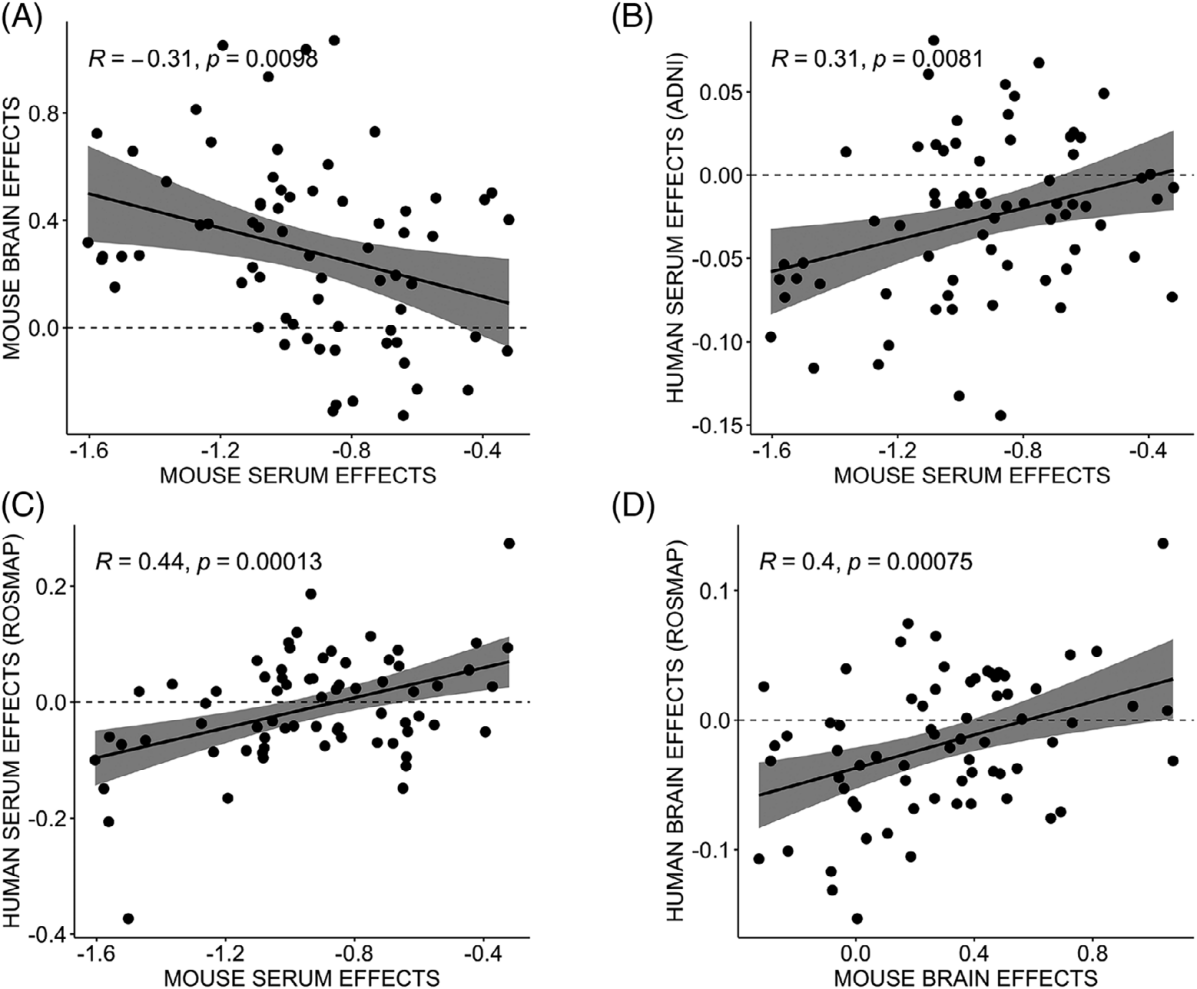
Correlation between human and mouse metabolomic signatures in *APOE* ε4 carriers. *APOE* ε4 effects on glycerophospholipids levels were measured between mouse and human metabolomes with Pearson correlation (*R*) and statistical significance. Each point is an effect estimate for a single glycerophospholipid species. A, Mouse serum and brain effects showing significant negative correlation in LOAD1 mice. B, ADNI human and mouse serum effects showing significant positive correlation. C, ROS/MAP human and mouse serum effects showing significant positive correlation. D, ROS/MAP human and mouse brain effects showing significant positive correlation. ADNI, Alzheimer's Disease Neuroimaging Initiative; *APOE*, apolipoprotein E; ROS/MAP, Religious Orders Study Rush Memory and Aging Project.

Upon comparing these brain associations to associations in the serum metabolome, we observed that out of 65 metabolites associated with 5XFAD in the brain metabolome, only 3 metabolites were associated with serum in 5XFAD, while 43 were associated with serum in LOAD1. This suggests that AD‐related effects present in different tissues in the different mouse models. Similarly, out of 12 metabolites associated with LOAD1 in the brain, 10 were associated with LOAD1 and 2 were associated with 5XFAD in serum. Metabolites commonly associated with either genotype across serum and brain metabolomes were generally glycerophospholipids and sphingolipids. Some amino acids such as valine and isoleucine were significantly associated with the LOAD1 genotype in serum metabolome with larger effect size in males (Figure 3C‐D, Table S3), while two amino acids (lysine and arginine) and five biogenic amines (spermidine, spermine, creatinine, putrescine, and t4‐OH‐Pro) were specifically significantly associated with 5XFAD in brain metabolome. Notably, levels of the significantly associated metabolites were higher in the brain metabolome and reduced in the serum metabolome compared to respective controls. We compared the effect of LOAD1 on glycerophospholipids (the largest class of metabolites in panel) levels for serum and brain metabolomes and observed a significant negative correlation (*r* = −0.31, *P* = 0.01) between tissues (Figure 4A).

### 3.4 Comparison of mouse metabolome profile to human metabolome study

Next, we compared our results to human metabolomic profiles from two independent studies: (1) the ADNI cohort, for which we have serum metabolomics data from 1517 participants^12^ and (2) the ROS/MAP,^31^ for which we had p180 metabolites data from both serum and brain from 92 participants.

### 3.5 The ADNI cohort

We investigated whether serum metabolites reported to be significantly associated with AD biomarkers in the ADNI cohorts^12^ were also associated with AD risk factors in mouse serum metabolome. Arnold et al.^12^ used multivariable logistic regression to measure associations of pathological Aβ_1‐42_ with metabolite concentrations as explanatory variables. For uniformity with our mouse data analysis, we re‐assessed the association analysis for the ADNI human serum metabolomics data using a multivariable linear regression approach using metabolite concentrations as the dependent variable to test associations of the CSF Aβ_1‐42_ pathology with concentrations of 139 serum metabolites. We recovered the same metabolites significantly associated with CSF Aβ_1‐42_ pathology as reported in the study.^12^

**FIGURE 5:**
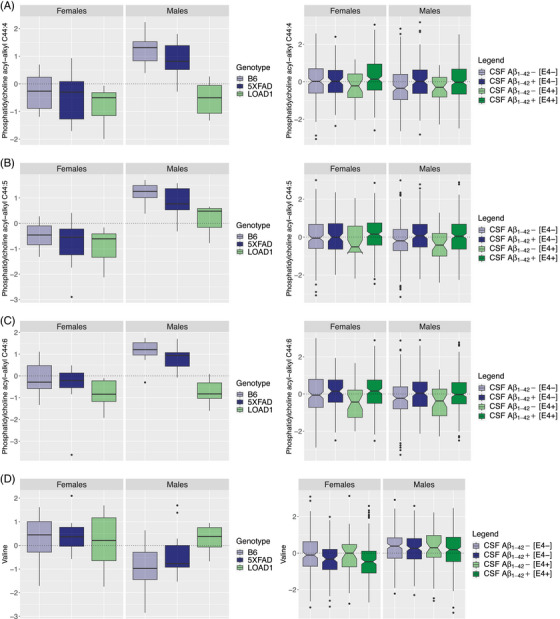
Levels of metabolites significantly associated with CSF Aβ_1‐42_ pathology in humans and LOAD1 genotype in mouse models. Boxplots showing levels for serum metabolites significantly associated with both LOAD1 genotype in mouse models (left panels) and CSF Aβ_1‐42_ pathology in human subjects (right panels). In humans, subjects were stratified by sex, *APOE* ε4 carriers, and CSF Aβ_1‐42_ pathology and mouse models were stratified by sex and genotypes. In left panels the mouse model's genotype specificity is illustrated by a color scale (light blue: B6, dark blue: 5XFAD, light green: LOAD1). In right panels stratification is illustrated by a color scale (light blue: without CSF Aβ_1‐42_ pathology without *APOE* ε4 carriers, dark blue: CSF Aβ_1‐42_ pathology without *APOE* ε4 carriers, light green: *APOE* ε4 carriers without CSF Aβ_1‐42_ pathology, dark green: *APOE* ε4 carriers with CSF Aβ_1‐42_ pathology). Levels of (A) phosphatidylcholine acyl‐alkyl C44:4 (PC ae C44:4); (B) phosphatidylcholine acyl‐alkyl C44:5 (PC ae C44:5); and (C) phosphatidylcholine acyl‐alkyl C44:6 (PC ae C44:6). Levels of these metabolites are reduced in serum of LOAD1 mice (left panels) (that carry the ε4 variant but do not exhibit amyloid plaques) as well as in serum of *APOE* ε4 carriers in humans (without CSF Aβ_1‐42_ pathology) (right panels). D, Levels of valine are increased in serum of LOAD1 male mice (left panel) in a similar fashion to *APOE* ε4 carriers in humans without CSF Aβ_1‐42_ pathology (right panel). Note that mouse data are identical to those in Figure 3C. Aβ, amyloid beta; *APOE*, apolipoprotein E; CSF, cerebrospinal fluid.

Interestingly, we observed that four metabolites significantly associated with CSF Aβ_1‐42_ pathology (PC ae C44:4, PC ae C44:5, PC ae C44:6, and valine) were also significantly associated with the LOAD1 genotype (Table S3). Three out of these four metabolites (PC ae C44:4, PC ae C44:5, and PC ae C44:6) also showed Bonferroni‐significant associations with pathological CSF Aβ_1‐42_ in *APOE* ε4 carriers.^12^ Further, we noticed a similar decrease in these metabolite levels in serum of *APOE* ε4 carriers (without CSF Aβ_1‐42_ pathology), as we have seen in LOAD1 mice that carry the ε4 variant but do not exhibit amyloid plaques (Figure 5). However, none of the significant associations reported for pathological CSF Aβ_1‐42_ was significantly associated with the 5XFAD genotype in mouse serum metabolome.

Furthermore, we measured correlations between the effects of *APOE* ε4 on glycerophospholipid levels in serum metabolomics from the ADNI cohort and mouse models. We found a significant positive correlation (*r* = 0.31, *P* = 0.008) between the effects of *APOE* ε4 on glycerophospholipid levels in human and mouse serum, suggesting a similar decrease in the serum of *APOE* ε4 carriers compared to non‐carriers (Figure 4B).

### 3.6 ROS/MAP cohort

Next, we carried out multivariable linear regression analysis on ROS/MAP serum and brain metabolomics data using metabolite concentrations of glycerophospholipids as dependent variables to measure the effects of the *APOE* ε4 genotype versus other *APOE* alleles. We then assessed the correlation between *APOE* ε4 effects on glycerophospholipid levels in serum metabolomics data from ROS/MAP carriers and APOE ε4 mouse models (i.e., LOAD1 mice). We observed a significant positive correlation (*r* = 0.44, *P* = 0.0001) between the effects of *APOE* ε4 on glycerophospholipids levels in human and mouse serum, suggesting a similar decrease in the glycerophospholipids in serum of *APOE* ε4 carriers compared to non‐carriers (Figure 4C). We also measured the correlation between the effects of *APOE* ε4 on glycerophospholipids levels in brain metabolomics data from ROS/MAP and mouse model carriers and found a significant positive correlation (*r* = 0.40, *P* = 0.0008; Figure 4D), suggesting an increase in the same metabolites in the brain that was consistent across humans and mice. We also compared the effects of *APOE* ε4 on glycerophospholipids levels from human serum and brain metabolomes in the ROS/MAP cohort, but we did not observe a significant negative correlation (*r* = 0.11, *P* = 0.4) as we observed in mouse models. In summary, we observed the same effect directions in humans and mice from both serum and brain metabolites when compared separately, while human serum‐to‐brain comparisons may suffer from limited power.

## 4 DISCUSSION

### 4.1 Summary

In this study, we have systematically investigated alterations in abundances of 142 metabolites in the serum and brain metabolome of the 5XFAD amyloid mouse model and a more recently created LOAD1 (APOE4.Trem2^R47H^) mouse model with LOAD genetics. We assessed the sex differences for metabolic associations in each mouse model and investigated changes in sexual dimorphisms of metabolic levels compared to B6 control mice. A complex pattern of sex and genotype effects was observed, with the most significant effects occurring in the brain of 5XFAD mice and serum of LOAD1 mice. To assess the translational relevance of the mouse models, we compared our findings to recent metabolomic studies from two human cohorts.^12^, ^31^ We observed similar glycerophospholipid signatures in human and mouse *APOE* ε4 carriers in both brain and serum.

### 4.2 The two mouse strains exhibit distinct sex‐specific biology, both with potential relevance to dementia

Overall, we observed distinct sex differences for the two mouse strains. Significant sexual dimorphism of serum metabolic levels was observed in LOAD1 mice carrying the *APOE* ε4/ε4 variant. In the serum metabolome, we observed that most glycerophospholipids were significantly associated with males and showed elevated levels compared to females, while few sphingomyelins and amino acids were more abundant in females. Multiple serum metabolites with significantly higher levels in B6 (controls) females (specifically amino acids including valine and arginine) showed reduced levels in female mice carrying *APOE* ε4/ε4 (LOAD1) compared to their male counterparts, while other metabolites such as glycerophospholipids and some long chain acylcarnitines showed reduced levels in male mice carrying *APOE* ε4/ε4 compared to females. Brain metabolomes showed more significant sexual dimorphisms in 5XFAD mice and brain metabolites such as some biogenic amines, glycerophospholipids, and sphingomyelins that had greater abundance in B6 males showed higher levels in the 5XFAD females. This suggests that metabolic sex differences changed owing to presence of the *APOE* ε4/ε4 allele and 5XFAD transgene. However, amino acids such as alanine, serine, threonine, and tryptophan exhibited significant increased levels in both brain and serum metabolome of female mice compared to male mice in all three strains, while some glycerophospholipids were significantly elevated in the brain and serum metabolome of male mice compared to female mice in all strains. We note that all mice were 6 months of age, unlike the human samples from aged individuals, and that sex‐specific metabolite profiles may evolve differently with aging. These results suggest that even though both mouse models are intended for use in AD research, the two strains have different sex‐specific biology with potentially distinct relevance to dementia.

### 4.3 The LOAD1 mouse model is appropriate for the study of AD metabolite biology

Our analysis of both brain and serum metabolomes from each animal suggests distinct tissues of action for the two genetic constructs. Sex‐stratified metabolite associations with genotypes identified that serum metabolites were more significantly associated with LOAD1 genotype, while brain metabolites were more significantly associated with 5XFAD. These outcomes are potentially due to 5XFAD amyloid accumulation primarily affecting the brain while the *APOE* ε4/ε4 in the LOAD1 strain affects lipid metabolism, potentially throughout the body. Notably, we identified a heterogenous association of valine in the LOAD1 genotype, with reduced levels in female mice but increased levels in males. Studies have associated the reduced level of valine in serum with cognitive decline and brain atrophy in AD,^38^ and also suggested valine as a marker for increased female vulnerability to AD.^12^, ^38^ We also observed increased levels of biogenic amine putrescine, specifically in the brain of female 5XFAD. As significant increased levels of putrescine have been reported in brain tissue from AD patients,^39^ similar elevations were also observed in the APP/PS1 mice at 6 months of age,^26^ suggesting Aβ causes upregulation of polyamine uptake and increased ornithine decarboxylase activity, which leads to increased polyamine synthesis,^40^, ^41^ which in turn causes dysfunction of the N‐methyl‐D‐aspartate receptor leading to the neuronal excitotoxicity which occurs in AD.^42^ Two other biogenic amines (spermidine, spermine) also exhibit increased but insignificant level in the 5XFAD female brain. Pan et al.^26^ reported that putrescine precedes both spermidine and spermine in the biochemical pathway and observed significant increase in both spermidine and spermine in female brain at later time point. This suggests that our relatively young mice (6 months of age) may exhibit more significant changes in these biogenic amines at later ages. The metabolite asymmetric dimethylarginine (ADMA), which is an endogenous inhibitor of nitric oxide synthase, has been found to be higher in plasma from AD patients.^43^ Inhibition of endothelial nitric oxide synthesis by ADMA impairs cerebral blood flow, which may contribute to the development of AD.^43^ We also observed a significant positive association of ADMA with female LOAD1 in serum metabolome, suggesting that the LOAD1 mouse model is appropriate for the study of relevant AD biology.

### 4.4 The 5XFAD amyloidogenic mouse is a relevant model for acylcarnitine alterations in AD

We identified both female‐specific and heterogeneous increases in sphingomyelins in the brain metabolome of 5XFAD mice. Sphingomyelins are precursors for ceramide production and their accumulation suggests the induction of apoptosis, further driving neurodegeneration by increasing Aβ biosynthesis and promoting gamma‐secretase processing of amyloid precursor protein.^44^, ^45^, ^46^ Higher levels of sphingomyelins and glycerophospholipids in 6‐month‐old mouse brains are indicative of early neurodegeneration and loss of membrane functions. Acylcarnitines have important functions in the brain such as mitochondrial function, energetics, and neurotransmission and have been linked with AD‐related pathology.^12^, ^47^, ^48^ We observed a significant female‐specific association of higher levels of acylcarnitine C18 with the 5XFAD, suggesting sex‐specific accumulation of long‐chain fatty acids in females. Increased levels of C18 have been also previously reported in MCI patients with CSF Aβ_1‐42_ pathology.^47^ These findings suggest the 5XFAD amyloidogenic mouse as a relevant model for acylcarnitine alterations in AD.

### 4.5 Serum biomarkers are informative, but their effects in the brain cannot be directly extrapolated

Glycerophospholipids (PCs and LysoPCs) are the major class of complex lipids playing essential roles in neural membrane formation and intraneuronal signal transduction.^49^ We identified that serum levels of glycerophospholipids were reduced in LOAD1 mice compared to B6 controls, while levels of these metabolites in the same animals were greater in both male and female brains of LOAD1 and 5XFAD mice. We also compared the LOAD1 genotype effects on glycerophospholipid levels between serum and brain metabolomes and observed a significant negative correlation. This suggests a correspondence between brain and serum glycerophospholipid levels, but a negative rather than positive correlation. Similar patterns of contrast concentration level of glycerophospholipids in brain and blood metabolome were also observed in the APP/PS1 mouse.^26^ These findings imply that although the serum biomarker is informative, effects in the brain cannot be directly extrapolated from those in blood.

### 4.6 The translational utility of mouse models for metabolomic studies of AD

We observed translational relevance for these results in two human AD studies. Our observed effect of LOAD1 on serum glycerophospholipids levels was significantly correlated with serum effects of the *APOE* ε4 variant in ADNI, showing a similar decrease in the same metabolites in serum of *APOE* ε4 carriers compared to non‐carriers and indicating a serum‐based effect of *APOE* genotype. We also identified Bonferroni‐significant associations of PC ae C44:4, PC ae C44:5, and PC ae C44:6 with *APOE* ε4 genotype in mouse models as in the ADNI cohort with consistent effect directions. While all three PCs showed homozygous associations in the ADNI cohort, in mice only PC ae C44:5 showed a homogeneous effect, while associations of PC ae C44:4 and PC ae C44:6 were male specific. We also confirmed tissue‐specific effects of the LOAD1 genotype on metabolite levels for *APOE* ε4 carriers in the ROS/MAP cohort, finding significant correlations between carriers and LOAD1 mice in both brain and serum metabolome. Altogether, this suggests similar effects of the *APOE* ε4 variant in mouse models to those observed in human AD carriers. However, we did not observe consistent sex differences between mice and humans. Contrary to the mouse models, PCs were higher in female humans and amino acids were (with few exceptions like serine and glycine) higher in males.^12^ Despite these dissimilarities of some sex differences, we still observed an overlap in associations with mouse genotype and the related human phenotypes, which indicates that the sex differences do not mask associations on the phenotypic/genotypic level. This suggests that, while there is a difference in the metabolic patterns for sex in mice and humans, there is translational utility in mouse models for metabolomic studies of AD.

## 5 CONCLUSION

In conclusion, metabolomic signatures were notably different between brain and serum in two mouse models of AD at 6 months of age. The early‐onset 5XFAD mice exhibited stronger effects in brain, whereas the late‐onset LOAD1 mouse showed more pronounced effects in serum. These findings are consistent with the high levels of neuropathology in 5XFAD mouse brains and the modifications of serum biomarkers in LOAD1 mice. We were able to identify patterns of metabolic changes related to human AD risk in our mouse models. These findings strengthen the utility of mouse models for studying metabolic changes that occur in human AD at early stages. However, there are some limitations in this study. First, we evaluated metabolomic data from only 6‐month‐old mice, which did not allow to study of progressive metabolic changes associated with AD. Studies using transgenic APP/PS1 mice observed that metabolic changes associated with AD pathology appeared first in the brain and later in blood.^26^ Therefore, it will be interesting to investigate metabolomics profiles of the LOAD1 and other late‐onset mouse models over different extended ages to study progressive metabolic changes associated with AD pathology. We have also used a targeted approach, potentially missing broader alterations in mouse model metabolomes. Our findings suggest such aging studies can model the different phases of metabolic disturbances which may occur in human AD.

## CONFLICT OF INTEREST STATEMENT

MA and RKD are co‐inventors (through Duke University/Helmholtz Zentrum München) on patents on applications of metabolomics in diseases of the central nervous system. RKD hold equity in Metabolon Inc., Chymia LLC, and PsyProtix. GWC has consulted for Astrex Pharmaceuticals. RSP, RB, JK, KPK, DG, HW, MS, and GRH have no conflicts to disclose. Author disclosures are available in the supporting information.

## CONSENT STATEMENT

No consent was required as all human subjects data were reused under controlled access with an active AD Knowledge Portal Data Use Certificate (v7.3). All data were anonymized by original sources with no possibility of deanonymization.

## Supporting information

Supporting Information

Supporting Information

Supporting Information

Supporting Information

Supporting Information

## Data Availability

Metabolomics datasets are available via the AD Knowledge Portal (https://adknowledgeportal.org). The AD Knowledge Portal is a platform for accessing data, analyses, and tools generated by the Accelerating Medicines Partnership (AMP‐AD) Target Discovery Program and other National Institute on Aging (NIA)‐supported programs to enable open‐science practices and accelerate translational learning. The data, analyses, and tools are shared early in the research cycle without a publication embargo on secondary use. Data are available for general research use according to the following requirements for data access and data attribution (https://adknowledgeportal.org/DataAccess/Instructions). For access to content described in this manuscript see: Jax.IU.Pitt Metabolomics (p180): https://www.synapse.org/#!Synapse:syn22313586. ADNI p180: https://www.synapse.org/#!Synapse:syn7440346. ROS/MAP p180: https://www.synapse.org/#!Synapse:syn26007829.
